# Randomized clinical trial: Effective gluten degradation by *Aspergillus niger*-derived enzyme in a complex meal setting

**DOI:** 10.1038/s41598-017-13587-7

**Published:** 2017-10-12

**Authors:** Julia König, Savanne Holster, Maaike J. Bruins, Robert J. Brummer

**Affiliations:** 1 Nutrition-Gut-Brain Interactions Research Centre, Faculty of Health and Medicine, School of Medical Sciences, Örebro University, Örebro, Sweden; 2 DSM Biotechnology Centre, Delft, Netherlands

## Abstract

The *Aspergillus niger*-derived prolyl endoprotease (AN-PEP) has previously been shown to degrade gluten in healthy subjects when added to an intragastrically infused meal. The current study investigated the efficacy of AN-PEP in a physiological meal setting. In this randomized placebo-controlled crossover study, 18 gluten-sensitive subjects consumed a porridge containing 0.5 g gluten together with two tablets either containing a high or low dose of AN-PEP, or placebo. Gastric and duodenal content was sampled over 180 minutes, and areas under the curve of gluten concentrations were calculated. The primary outcome, i.e. success rate of high dose AN-PEP defined as at least 50% gluten degradation compared to placebo in the duodenum, was achieved in 10 of 13 comparisons. In the stomach, gluten levels were reduced from 176.9 (median, interquartile range 73.5–357.8) to 22.0 (10.6–50.8, p = 0.001) in the high dose and to 25.4 μg × min/ml (16.4–43.7, p = 0.001) in the low dose. In the duodenum, gluten levels were reduced from 14.1 (8.3–124.7) in the placebo to 6.3 (3.5–19.8, p = 0.019) in the high dose and to 7.4 μg × min/ml in the low dose (3.8–12.0, p = 0.015). Thus even in a physiological meal setting, AN-PEP significantly degraded most gluten in the stomach before it entered the duodenum.

## Introduction

The consumption of gluten proteins found in wheat and of related proteins found in other grains causes serious problems for individuals suffering from gluten-related disorders. Gluten is characterized by a high abundance of glutamine and proline (15%)^1^. Proline residues are not accepted by most potential cleavage site of proteases^2^, which makes proline-rich gluten proteins resistant to the digestion by enzymes of the human gastrointestinal tract. When these proteins reach the lamina propria of the small intestinal mucosa, they are modified by tissue transglutaminase, resulting in higher affinity for HLA-DQ2 and HLA-DQ8 molecules on antigen-presenting cells. In coeliac disease, this leads to an abnormal immune reaction that can ultimately result in villous atrophy and malabsorption^3,4^.

The immunogenicity of gluten could be reduced by breaking down gluten into smaller peptides^3^. In this context, several dietary digestive enzyme supplements can be found on the market that claim to degrade gluten. Most of these products contain the fungal enzyme DPPIV, a dipeptidyl peptidase. DPPIV has its pH optimum at a neutral pH, suggesting that it might not be very effective in the low pH environment of the stomach. In addition, DPPIV only releases proline-containing dipeptides from the N-terminus and has no endoprotease activity, so that larger, polypeptide remnants still may contain immunogenic activity^5^. In a recent *in vitro* study, only one of the tested enzyme supplements was able to effectively degrade the immunogenic epitopes of gluten in the pH range of the stomach^6^. The *Aspergillus niger*-derived prolyl endoprotease (AN-PEP) successfully cleaved those epitopes into smaller, non-immunogenic peptides of eight amino acids or smaller^6^.

AN-PEP is active between a pH of 2 and 8, with an optimal activity between pH 4 and 5. It is not degraded by pepsin, thereby remaining fully functional in the stomach^7^. It specifically degrades gluten epitopes by cleaving behind proline residues. In a multi-compartmental gastrointestinal *in vitro* model, AN-PEP was able to degrade almost all gluten in the stomach compartment before it reached the small intestinal compartment^8^. In a subsequent study by Salden *et al*., 12 healthy volunteers received either a low or high calorie liquid meal containing 4 g of gluten together with either AN-PEP or placebo in a crossover design. AN-PEP efficiently degraded gluten in the stomach of the healthy subjects, irrespective of the caloric density of the meal and thus the gastric emptying rate^9^.

The aim of the current study was to test the efficacy of AN-PEP to degrade gluten when administered in tablet form in a complex physiological meal setting, in which gluten was present in a small amount, representing hidden or residual gluten. Surveys have shown that subjects avoiding gluten are still exposed to approximately 150 mg/d of gluten on a non-strict diet^10,11^ and to 7 mg/d on a strict gluten-free diet^11^. In the current study, we included self-reported non-coeliac gluten-sensitive subjects as a target group for the use of AN-PEP to reduce unwanted gluten exposure. Coeliac disease patients should be very strict in gluten avoidance and were therefore not considered to be an appropriate study population in this placebo-controlled study. Gluten-sensitive subjects suffer from symptoms similar to those of coeliac disease patients, including intestinal complaints such as diarrhoea, constipation, and bloating, as well as extra-intestinal symptoms such as chronic fatigue^12,13^. However, subjects are negative for coeliac disease specific anti-tissue transglutaminase antibodies and their duodenal epithelial mucosa appears to be intact^14^.

In our study, AN-PEP was administered to 18 self-reported gluten-sensitive subjects in tablet form in two different doses together with a meal containing 0.5 g of gluten, in a randomized, placebo-controlled, crossover fashion.

## Results

### Baseline characteristics of subjects and adverse effects

Eighteen subjects were enrolled in the study and randomized, all fulfilling the in- and exclusion criteria. In total, n = 16 completed all three test days and n = 2 subjects dropped out after the first test day. One of these two subjects discontinued the study due to discomfort related to placement of the nasoduodenal tube (received the high dose AN-PEP), the other participant discontinued to health problems unrelated to the study (received the low dose AN-PEP). Results from those two subjects were excluded from efficacy analyses in which comparison to placebo was required. Subject characteristics from all subjects enrolled as well as excluding drop-outs are presented in Table 1. For a participant flow chart see Supplemental Fig. 1.

**Table 1:** Baseline characteristics of study participants.

	All enrolled subjects	Excluding drop-outs
Female/Male	10/8	9/7
Age (mean ± SD)	25.6 ± 4.0	25.8 ± 4.2
BMI (kg/m^2^, mean ± SD)	23.9 ± 5.0	24.2 ± 5.2
Duration of gluten-sensitivity (unknown/0.5–1 yr/1–5 yrs/>5 yrs)	1/1/9/7	1/1/8/6

**Figure 1:**
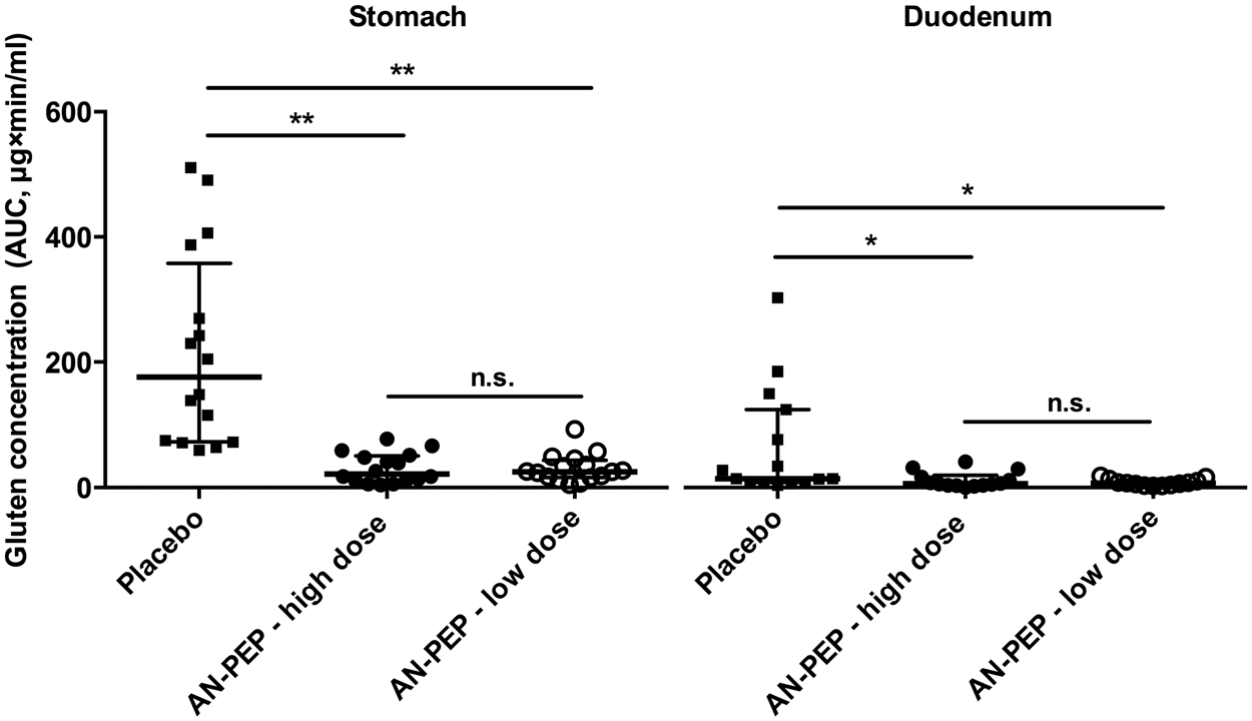
AUC_0–180_ values of gluten concentrations in the stomach and duodenum. Individually plotted scores for AUC_0–180_ values together with bars in black indicating median and interquartile ranges are shown. Both the high dose and the low dose AN-PEP significantly lowered the gluten concentrations in the stomach and in the duodenum, compared to the placebo administration. Significance was assessed using a non-parametric Wilcoxon signed-ranked paired test at α = 0.05 with a Bonferroni correction. **p < 0.01, *p < 0.05.

The difficulty in sampling fluid from the stomach and especially the duodenum resulted in missing values for the Area under the curve (AUC) calculations of the gluten concentrations in some participants. The missing values were equally distributed between the different test days, i.e. independent of if treatment or placebo was consumed.

From the stomach samples, the following number of test days were excluded: High dose AN-PEP: n = 2, low dose: n = 2, placebo: n = 2. From the duodenum samples, the following number of test days were excluded: High dose AN-PEP: n = 4, low dose: n = 5, placebo: n = 3. The following comparisons were available: High dose versus placebo stomach: n = 15; low dose versus placebo stomach: n = 15; high dose versus placebo duodenum: n = 13; low dose versus placebo duodenum: n = 12.

Three study participants reported n = 4 mild adverse events, of which n = 3 were rated as ‘not related to study product’, and n = 1 was rated as ‘suspected relation to study product’. No severe adverse events were reported.

### Efficacy of AN-PEP in degrading gluten

#### Interim analysis

The interim analysis after n = 5 volunteers who completed three test days showed a minimum of two successes (defined as at least 50% gluten degradation compared to placebo, calculated using 180 min AUC) in the low-dose arm. Hence, the low-dose arm was continued.

#### Primary and secondary endpoints (success rates)

The primary outcome was defined as the efficacy of the high dose AN-PEP in degrading gluten based on the amount of gluten detected in the duodenum compared with placebo over 180 min. Success was defined as at least 50% gluten degradation compared to placebo, calculated using AUC. Of the n = 13 comparisons available, n = 10 were successes. Using an exact binomial confidence interval of 95%, this data suggests that the minimum probability of success rates to be expected is 51%. The low dose was successful in n = 7 out of n = 12 available comparisons in the duodenum, suggesting a minimum probability of successes of 32%. In the stomach, the high dose was successful in n = 12 out of n = 15 available comparisons, suggesting a minimum probability of successes of 56%, whereas the low dose was successful in n = 13 out of n = 15 available comparisons, suggesting a minimum probability of successes of 64% (see Table 2).

**Table 2:** Success rates and binomial 95% confidence intervals of high and low dose AN-PEP compared to placebo.

	Stomach		Duodenum	
	High dose	Low dose	High dose	Low dose
n (successes^1^)/n (available comparisons)	12/15	13/15	10/13	7/12
Actual success rate (%)	80.0	86.7	76.9	58.3
Binomial 95% confidence interval (lower bound, %)	56.0	63.7	50.5	31.5

^1^Success was defined as at least 50% gluten degradation compared to placebo, calculated using the 180 min Area Under the Curve (AUC).

#### Average reduction in gluten concentrations (continuous measure)

Both the high dose and the low dose AN-PEP significantly lowered the gluten concentrations (AUC_0–180min_) in the stomach as well as in the duodenum compared to the placebo (see Fig. 1). In the stomach, gluten levels were reduced from a median of 176.9 μg × min/ml in the placebo (interquartile range 73.5–357.8) by 88% to 22.0 μg × min/ml (10.6–50.8, p = 0.001) in the high dose and by 86% to 25.4 μg × min/ml (16.4–43.7, p = 0.001) in the low dose, respectively. In the duodenum, gluten levels were reduced from 14.1 μg × min/ml (8.3–124.7) in the placebo by 56% to 6.3 μg × min/ml (3.5–19.8, p = 0.019) in the high dose and by 48% to 7.4 μg × min/ml (3.8–12.0, p = 0.015) in the low dose, respectively.

**Figure 2:**
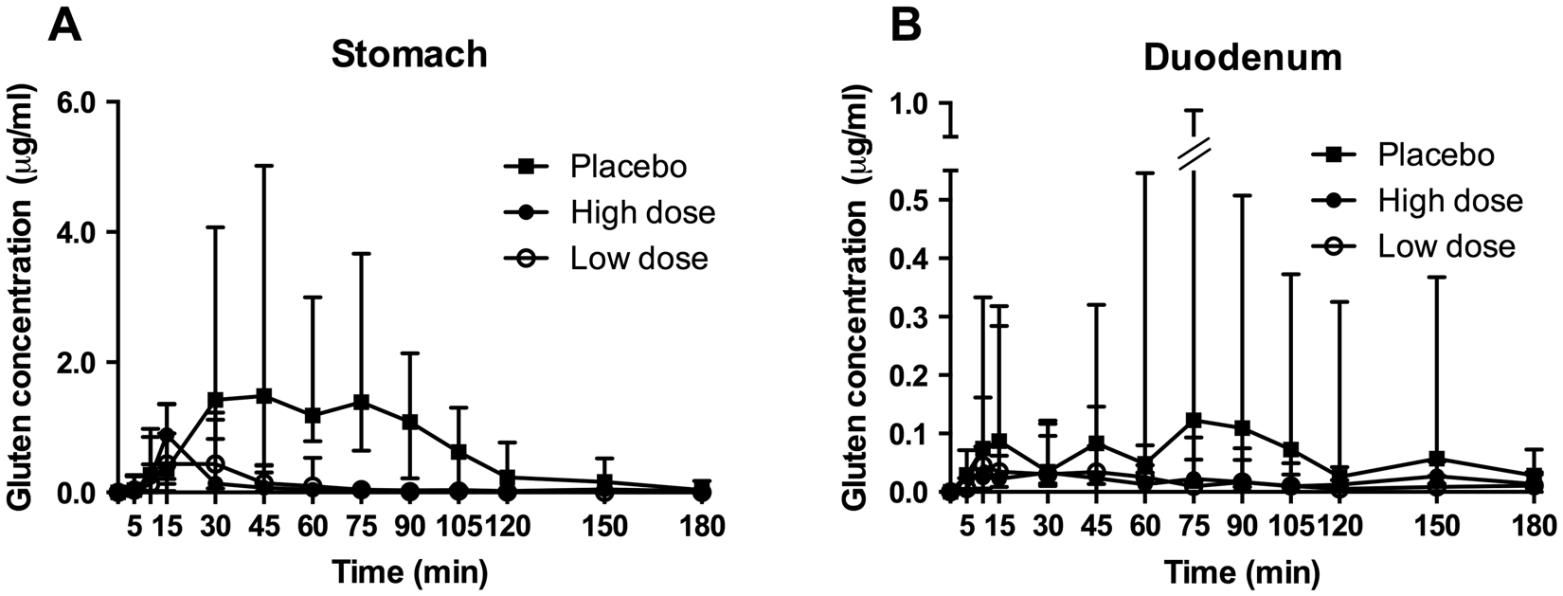
Gluten concentrations in the stomach (**A**) and duodenum (**B**) over time. Median values and interquartile ranges of gluten concentrations measured at different time points are shown. The y-axis in (**B**) is intercepted for visibility reasons and interquartile ranges below zero are not shown.

The median values of gluten concentrations in the stomach and duodenum over time are shown in Fig. 2. The individual values of gluten concentrations in the stomach and duodenum over time are shown in Supplemental Figs 2 and 3, respectively.

Post-hoc analyses showed no significant differences between the efficacy of the low and the high dose of AN-PEP.

## Discussion

The results from this double-blinded, placebo-controlled study showed that both a low and a high dose of AN-PEP in the form of tablets significantly reduced the gluten concentrations in the stomach and duodenum of gluten-sensitive individuals. These results confirm the findings of Salden *et al*. who assessed the efficacy of AN-PEP in degrading gluten in the stomach of healthy volunteers. In the latter study, the gluten-containing meal (4 g of gluten) was administered in liquid form into the stomach via a gastric feeding tube. In our study, gluten was provided as part of a complex, physiological meal in an amount of circa 0.5 g. We chose this rather low amount of gluten to represent the amount of gluten that gluten-intolerant subjects might unintentionally consume even when trying to avoid gluten^10,11^. In addition, while Salden *et al*. administered AN-PEP in liquid form, we chose to assess the efficacy of the enzyme in tablet form and in a solid meal, as this reflects daily practice. Salden *et al*. aimed to maximize the efficacy of the enzyme by mixing AN-PEP in liquid form into a liquid meal. Nevertheless, even under the conditions of our study, where AN-PEP was provided in the form of solid, fast-dissolving tablets, the efficacy in degrading gluten was comparable to the previous study.

It is important to emphasize that this study was designed to evaluate the efficacy of AN-PEP in degrading gluten when provided as a tablet together with a complex meal. Evaluating the effect of AN-PEP on gluten-induced clinical symptoms was beyond the scope of this study, as the small amount of gluten given on three separate occasions would be unlikely to cause significant symptoms. The latter had been intended by a clinical trial by Tack *et al*., in which 14 coeliac disease patients were randomized to gluten intake (7 g/d) with either AN-PEP or placebo for two weeks^15^. However, even in the placebo group, this rather large amount of gluten did not lead to clinical deterioration in the enrolled coeliac disease patients, thus an effect of AN-PEP on clinical markers and symptoms could not be shown. Although not designed as a safety study, no severe or serious adverse events were reported in this study, and the number of reported gastrointestinal complaints did not differ between the AN-PEP and the placebo group^15^. Our study was not designed to evaluate safety of the enzyme, as this would require longer exposure and exclusion of other intervention components such as gluten intake and placement of the nasoduodenal tube. Study participants consumed the test product only on three occasions, separated by at least one week of wash-out period.

To prevent unnecessary exposure to the relatively invasive nasoduodenal tube placement and related risk of dropout in this crossover design, we chose to perform an interim analysis, by an external board, to allow for early termination of a potential non-effective AN-PEP dose. The results, however, showed that also the low dose, based on n = 5 participants, substantially reduced gluten concentrations in both the stomach and the duodenum.

Results from previous *in vitro* experiments indicate that the effect of AN-PEP on gluten degradation is dose-dependent^16^. However, in this clinical study, post-hoc analyses showed no significant difference between the efficacy of the low and the high dose of AN-PEP, and hence it is difficult to conclude if there is a dose-dependent effect. Possibly, under the current study conditions, the optimal enzyme-to-gluten ratio was already achieved by the low dose enzyme.

In our study, we included self-reported non-coeliac gluten-sensitive individuals as test subjects. It is still controversial if it is actually gluten that causes the symptoms in these subjects. While some studies showed that a gluten-free diet could improve symptoms in non-coeliac gluten sensitive individuals^17,18^, other studies did not confirm this^19,20^. As other compounds in wheat instead of gluten might be involved in causing symptoms, the use of the term non-coeliac *wheat* sensitivity has been proposed instead^19,20^.

To date, no objective clinical diagnostic criteria or biomarkers are available for the diagnosis of non-coeliac gluten or wheat sensitivity, even though advances are being made in understanding the mechanisms behind. These possibly involve a systemic immune activation in combination with a comprised intestinal barrier function^21^. We identified gluten-sensitive individuals based on self-reported symptoms when consuming gluten and by exclusion of coeliac disease and wheat allergy with high likelihood (negative serological tests for anti-tissue transglutaminase IgA antibodies and wheat protein IgE antibodies, respectively). We did not perform a double-blinded placebo-controlled gluten challenge as sometimes suggested to confirm the diagnosis of gluten sensitivity, which could be regarded as a limitation of the study. However, we considered this dispensable, as there is no evidence that the physiology of gluten degradation by an enzyme would differ between healthy and gluten intolerant subjects, and as we did not evaluate the effect of AN-PEP on symptom improvements in this study.

We did not sample duodenal biopsies to confirm absence of coeliac disease. Most of the participants (15 out of 18) stated that they occasionally consumed gluten-containing foods, which increases the probability that we would have identified participants with coeliac disease due to increased anti-tissue transglutaminase IgA antibody serum levels. Even though we cannot totally exclude the possibility that some of study participants suffered from coeliac disease instead of non-coeliac gluten-sensitivity, the low and temporary amount of gluten used in our study should have been safe even for those participants and would not justify a duodenoscopy in all subjects.

In conclusion, our study showed that the AN-PEP enzyme is effective in degrading small amounts of gluten as part of a complex meal in the stomach. Even though the use of AN-PEP is not intended to replace a gluten-free diet in gluten-related disorders, it appears to be effective as a digestive aid protecting against the unintentional intake of gluten.

## Methods

The study was conducted according to the principles of the Declaration of Helsinki and its revisions, and ethical approval was obtained from the Central Ethical Review Board of Uppsala, Sweden (registration number 2013/479). The study was performed at Örebro University Hospital in Örebro, Sweden from September 2014 until June 2015. All participants were recruited in the greater area of Örebro and gave their written informed consent before participation. The trial has been registered at ClinicalTrials.gov (NCT02060864) on February 11, 2014.

### Subjects

Subjects with self-reported gluten sensitivity but otherwise healthy were recruited by advertisement at Örebro University and Örebro University Hospital. Inclusion criteria were self-reported symptoms when consuming gluten, an age between 18 and 70 years and the use of contraceptive agents for women. Reasons to exclude subjects were coeliac disease serology (positive serological test for tissue transglutaminase IgA antibodies and total IgA antibodies), wheat allergy (positive serological test for wheat protein IgE antibodies), intake of medication affecting gastric emptying or secretion (except oral contraceptives), pregnancy or breast-feeding, inability to swallow a gastroduodenal catheter, and any medical condition likely to interfere with the study.

### Sample size calculations

Sample-size calculations indicated that a sample size of 11 participants would have 87% power to detect a difference between the active preparation and placebo of 69% (70% vs. 1%), expecting the proportion of discordant pairs to be 0.71, and the method of analysis being an exact binomial sign test for paired proportions with a 5% two-sided significance level. The sample size estimation was performed with NQuery Advisor (Statistical Solution, Cork, Ireland). To account for unexpected technical failures and an estimated drop-out rate of 30–40%, a total of n = 18 participants were planned to be included.

### Study design and intervention

In this double-blinded, placebo-controlled, randomized crossover study, participants attended three test days with a one-week washout period in between. Subjects were randomized in a double-blind fashion to placebo, low-dose AN-PEP or high-dose AN-PEP. The randomization list was generated by an external party (Metronomia GmbH, Germany) using the SAS PROC PLAN procedure (SAS system 9.1.3). All participants and investigators remained blinded until all analyses were completed. Subjects attended each test day after an overnight fast, and a multi-lumen nasoduodenal catheter was placed with one lumen tip in the gastric antrum and one lumen tip 15 cm lower in the duodenum using the CORTRAK 2 Enteral Access System (CORPAK Medsystems, Buffalo Grove, Illinois, U.S.A.). Correct positioning of the catheter was confirmed by measuring pH values of the aspirates and continuous visual monitoring of the catheter guide wire by the CORTRAK device. After catheter placement, subjects consumed an oat porridge to which approximately 0.5 g gluten in the form of two crumbled wheat cookies were added. Two tablets providing no ANPEP (placebo), low-dose AN-PEP, or high-dose AN-PEP were given at the start of the consumption of the porridge. Stomach and duodenal content (0.5 to 2 ml) was sampled by aspiration before (−15 min) and at 5, 10, 15, 30, 45, 60, 75, 90, 105, 120, 150 and 180 minutes after consumption of the intervention. These time points were chosen as in a similar study by Salden *et al*., no gluten could be detected after 180 min^9^. The catheter was flushed with sterile saline after each aspiration. pH of each sample was measured and any residual enzyme activity in the samples was stopped by instantly increasing the pH to greater than 11 using 3 M NaOH and freezing the samples in liquid nitrogen. Samples were stored at −80 °C until analysis.

### Intervention

The AN-PEP enzyme and placebo tablets were provided by DSM Nutritional Products (Kaiseraugst, Switzerland) and manufactured by Aenova Group (München, Germany). The placebo tablets had the same appearance as the enzyme tablets. The tablets were packaged in carton boxes containing three blisters, one blister contained two tablets for each test day. The labels contained the subject ID and test day number, and did not contain any information about the intervention. The low dose tablets provided 83300 Protease Picomol International (PPI), and the high dose 166700 PPI of AN-PEP enzyme (1 PPI is the amount of enzyme that releases one picomole of p-nitroaniline per second under defined assay conditions).

The breakfast was prepared at a food-grade facility at the University Hospital Örebro shortly before the intervention. The standardized breakfast (~200 kcal) consisted of 20 g gluten-free oatmeal prepared with 60–75 g boiled water, 10 g margarine, 15 g jam, and two crumbled wheat cookies containing approximately 0.5 g of gluten. 200 ml of mineral water were provided for drinking. The participants consumed two spoons of the breakfast immediately before swallowing the tablets to prevent immediate passage from the stomach into the duodenum.

### Gluten extraction and analysis

The aspiration samples from the stomach and duodenum were heated at 85 °C for 10 min in order to inactivate any residual AN-PEP enzyme activity. 1 mL of UPEX solution (5 mM TCEP (646547, Sigma-Aldrich), 2% N-lauroylsarcosine (61745, Sigma-Aldrich) in PBS, pH 7) was added to 100 μl of sample and mixed thoroughly. The samples were incubated at 50 °C for 40 min. After adding 3 ml of 80% ethanol/water (v/v), the samples were mixed thoroughly and incubated for 1 hour at room temperature in a rotary shaker at 40 rpm. Then the samples were centrifuged at room temperature for 10 min at 2500 × g. After diluting the samples 1:2 in dilution buffer, the gluten epitope DQ2.5-glia-α3 (formerly called glia-α20) was quantified using the Gluten-Tec ELISA assay (EuroProxima B.V., Arnhem, The Netherlands) and then converted to the corresponding gluten content according to manufacturer’s instructions^22^. The limit of detection for this assay is specified with 2.5 µg/g gluten^22^.

### Statistical/analytical issues

The primary outcome was defined as the efficacy of the high dose AN-PEP in degrading gluten, based on the concentration of gluten detected in the duodenum compared to placebo. A success of AN-PEP in degrading gluten was defined as at least 50% gluten degradation compared to placebo, calculated as area under the curve (AUC) over 180 min. AUC was calculated using the trapezoid method (STATA release 14). Secondary outcomes were defined as the efficacy of low dose AN-PEP in degrading gluten based on concentration of gluten detected in the duodenum compared to placebo over 180 min, as well as efficacy of AN-PEP at either low or high dose in degrading gluten in the stomach. An additional secondary outcome was defined as the average reduction in gluten concentration (as a continuous measure) following administration of AN-PEP (low and high dose, respectively), defined as the percentage of gluten degraded compared to placebo over 180 min using AUC.

The lower bound of the exact binomial 95% confidence interval was used to infer a likely minimum probability for the expected number of successes^23^ (R version 3.2.4). The non-parametric Wilcoxon signed-ranked paired test at α = 0.05 with a Bonferroni correction was used to detect significant differences in gluten concentrations between treatment and placebo (calculated using AUC, SPSS version 23). These statistical analyses were defined before un-blinding, and no changes were made afterwards. After n = 5 volunteers had completed three test days, an interim analysis was performed by an independent third party (independent statistician at Örebro University) in order to decide whether to proceed with the low dose of AN-PEP. The decision to discontinue the low-dose arm was defined beforehand in the case of less than two successes (i.e. more than three failures) in the low-dose arm, as the efficacy would have been likely to be less than the minimum level specified before the trial.

### Missing values for AUC calculation due to technical/procedure-related problems

The sampling of gastric and especially duodenal fluids from all 13 time points (−15, 5, 10, 15, 30, 45, 60, 75, 90, 105, 120, 150 and 180 minutes) was difficult to achieve in some patients. As this could affect the accuracy of AUC calculations, the following requirement was defined regarding missing samples (time points) before un-blinding: Samples from at least 11 out of 13 time points were acceptable for an AUC calculation (corresponding to 15% missing values). As the AUCs generally reach their peak before 75 min, also the following pre-defined requirements were valid: Values from 10 out of 13 time points were acceptable if at least one of the missing values was at 120 min or later (for stomach samples, 150 min for duodenum samples); 9 out of 13 time points were acceptable if at least two of the missing values were at 120 min or later (for stomach samples, 150 min for duodenum samples). If there were too many missing values to reliably calculate the AUC for one test day, the data of this test day was excluded. Equal distribution of missing values between test days with the investigational product and the placebo was tested with Little’s MCAR (missing completely at random) test (SPSS version 23).

### Data availability

The datasets generated during and/or analysed during the current study are available from the corresponding author on reasonable request.

## Electronic supplementary material

Supplementary information

## References

[1] Wieser H (2007). Chemistry of gluten proteins. Food Microbiology.

[2] Bromme D, Bescherer K, Kirschke H, Fittkau S (1987). Enzyme-substrate interactions in the hydrolysis of peptides by cathepsins B and H from rat liver. Biochem J.

[3] Shan L (2002). Structural basis for gluten intolerance in celiac sprue. Science.

[4] Ludvigsson JF (2013). The Oslo definitions for coeliac disease and related terms. Gut.

[5] Yoshimoto T, Walter R (1977). Post-proline dipeptidyl aminopeptidase (dipeptidyl aminopeptidase IV) from lamb kidney. Purification and some enzymatic properties. Biochim Biophys Acta.

[6] Janssen G (2015). Ineffective degradation of immunogenic gluten epitopes by currently available digestive enzyme supplements. PLoS One.

[7] Stepniak D (2006). Highly efficient gluten degradation with a newly identified prolyl endoprotease: implications for celiac disease. Am J Physiol Gastrointest Liver Physiol.

[8] Mitea C (2008). Efficient degradation of gluten by a prolyl endoprotease in a gastrointestinal model: implications for coeliac disease. Gut.

[9] Salden BN (2015). Randomised clinical study: Aspergillus niger-derived enzyme digests gluten in the stomach of healthy volunteers. Aliment Pharmacol Ther.

[10] Hopman EG, le Cessie S, von Blomberg BM, Mearin ML (2006). Nutritional management of the gluten-free diet in young people with celiac disease in The Netherlands. J Pediatr Gastroenterol Nutr.

[11] Lovik A, Skodje G, Bratlie J, Brottveit M, Lundin KE (2017). Diet adherence and gluten exposure in coeliac disease and self-reported non-coeliac gluten sensitivity. Clin Nutr.

[12] Sapone A (2012). Spectrum of gluten-related disorders: consensus on new nomenclature and classification. BMC Med.

[13] Fasano A, Sapone A, Zevallos V, Schuppan D (2015). Nonceliac gluten sensitivity. Gastroenterology.

[14] Sapone A (2010). Differential mucosal IL-17 expression in two gliadin-induced disorders: gluten sensitivity and the autoimmune enteropathy celiac disease. Int Arch Allergy Immunol.

[15] Tack GJ (2013). Consumption of gluten with gluten-degrading enzyme by celiac patients: a pilot-study. World J Gastroenterol.

[16] Montserrat V, Bruins MJ, Edens L, Koning F (2015). Influence of dietary components on Aspergillus niger prolyl endoprotease mediated gluten degradation. Food Chem.

[17] Biesiekierski JR (2011). Gluten causes gastrointestinal symptoms in subjects without celiac disease: a double-blind randomized placebo-controlled trial. Am J Gastroenterol.

[18] Di Sabatino A (2015). Small Amounts of Gluten in Subjects With Suspected Nonceliac Gluten Sensitivity: A Randomized, Double-Blind, Placebo-Controlled, Cross-Over Trial. Clin Gastroenterol Hepatol.

[19] Biesiekierski JR (2013). No effects of gluten in patients with self-reported non-celiac gluten sensitivity after dietary reduction of fermentable, poorly absorbed, short-chain carbohydrates. Gastroenterology.

[20] Zanwar VG (2016). Symptomatic improvement with gluten restriction in irritable bowel syndrome: a prospective, randomized, double blinded placebo controlled trial. Intest Res.

[21] Uhde M (2016). Intestinal cell damage and systemic immune activation in individuals reporting sensitivity to wheat in the absence of coeliac disease. Gut.

[22] Mujico JR (2012). Validation of a new enzyme-linked immunosorbent assay to detect the triggering proteins and peptides for celiac disease: interlaboratory study. J AOAC Int.

[23] Clopper CJ, Pearson ES (1934). The Use of Confidence or Fiducial Limits Illustrated in the Case of the Binomial. Biometrika.

