# Supplementary material for: Randomized clinical trial: Effective gluten degradation by Aspergillus niger-derived enzyme in a complex meal setting
Source: Sci Rep. 2017 Oct 12;7:13100. doi: 10.1038/s41598-017-13587-7 (PMC5638938; doi:10.1038/s41598-017-13587-7)

SUPPLEMENTARY INFORMATION

Randomized clinical trial: Effective gluten degradation by *Aspergillus niger*-derived enzyme in a complex meal setting

Julia König[1], Savanne Holster[1], Maaike J Bruins[2], Robert J Brummer[1].

[1]Nutrition-Gut-Brain Interactions Research Centre, Faculty of Health and Medicine, School of Medical Sciences, Örebro University, Örebro, Sweden. [2]DSM Biotechnology Centre, Delft, Netherlands.

Changed affiliation: MJB changed affiliation from February 1, 2017: DSM Nutritional Products, Kaiseraugst, Switzerland.

Supplemental Figure 1 König et al.: CONSORT 2010 Flow Diagram

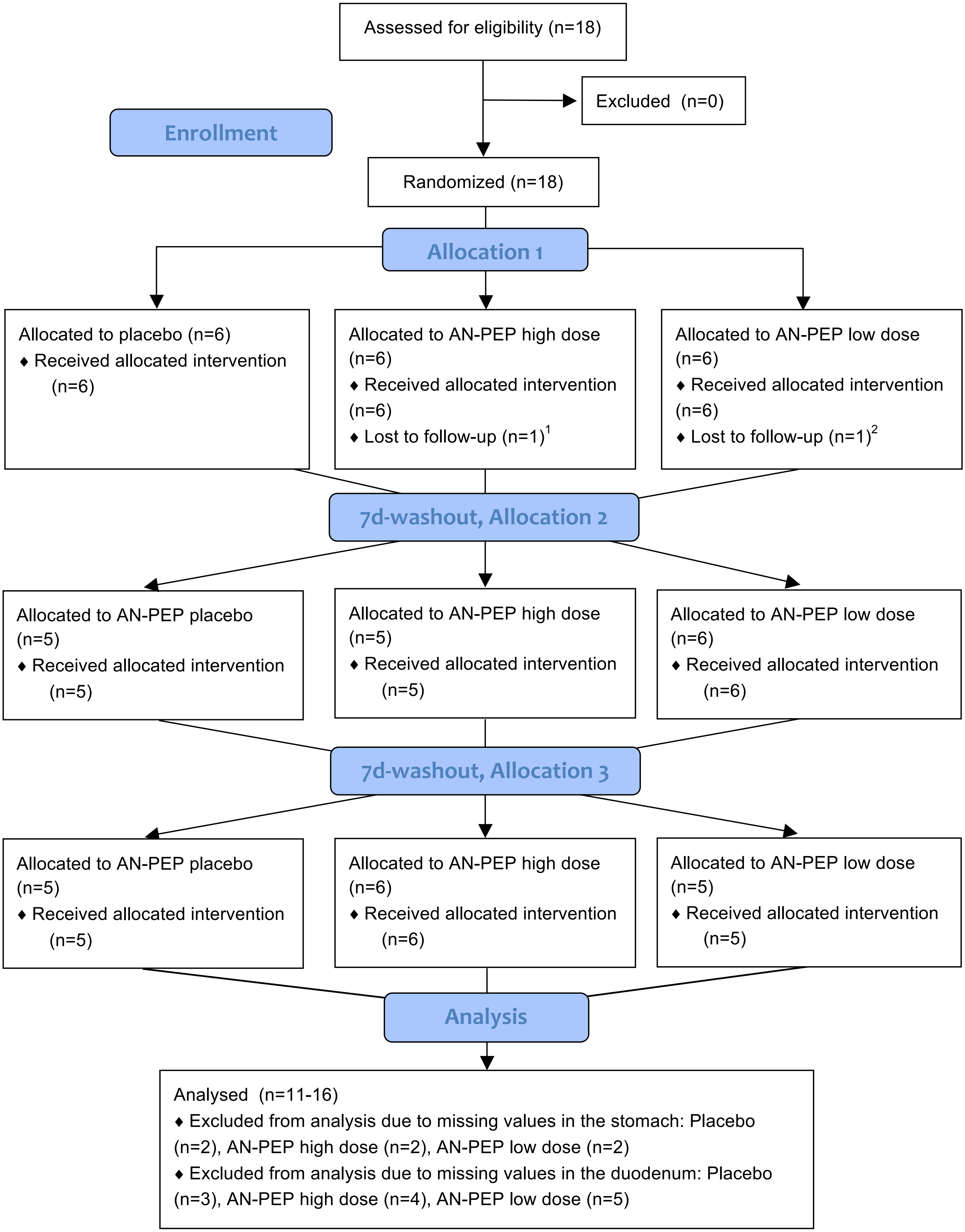

[1]*discomfort related to placement of nasoduodenal tube;* [2]*health problems unrelated to the study*

Supplemental Figure 2: Individual gluten concentrations (stomach)

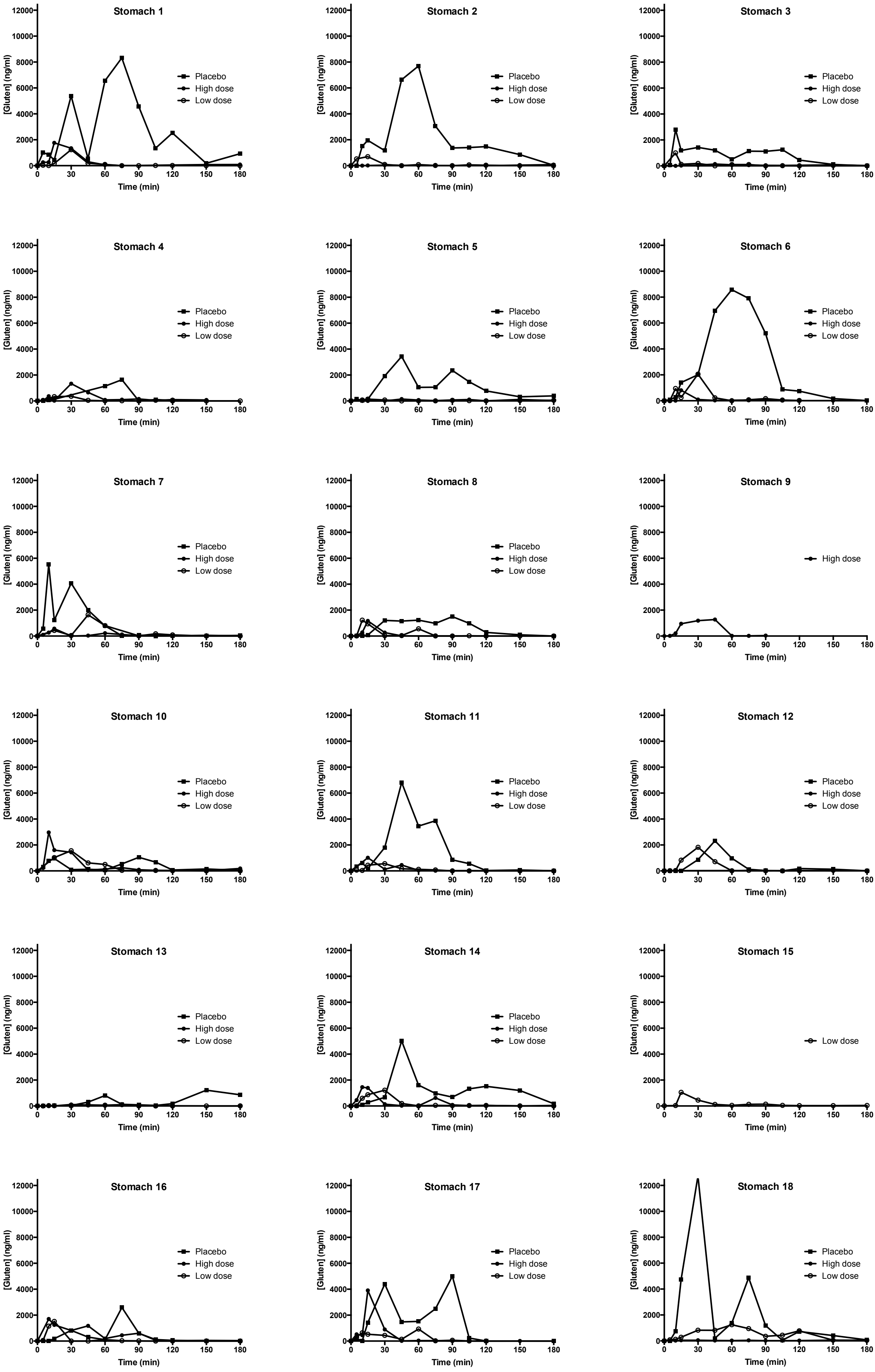

Supplemental Figure 3_Individual gluten concentrations (duodenum)

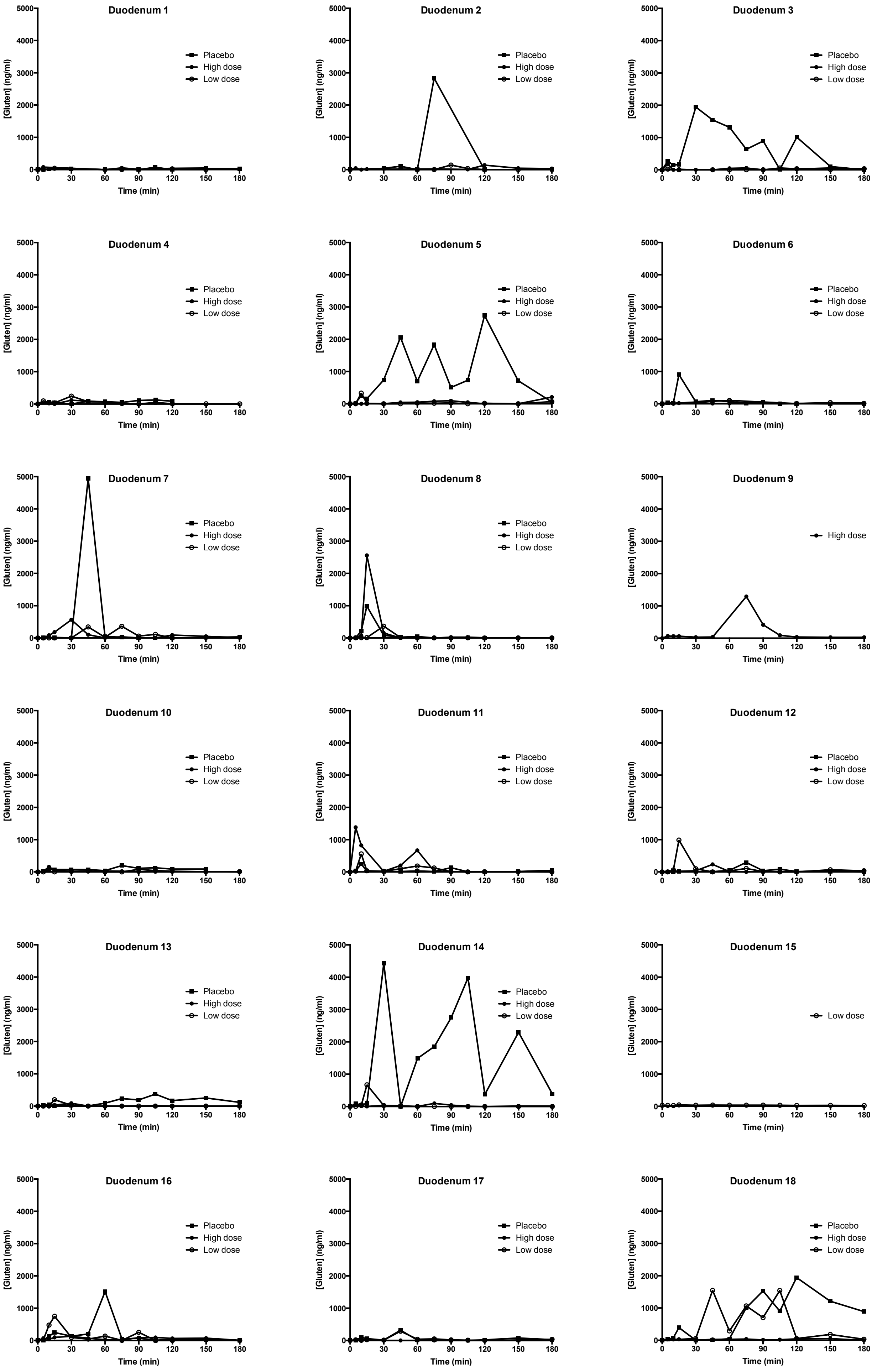

Supplement: Supplementary file 1 — Supplementary information [file 41598_2017_13587_MOESM1_ESM.pdf]
